# External Validation and Clinical Applicability of Two Optical Coherence Tomography–Based Risk Calculators for Detecting Glaucoma

**DOI:** 10.1167/tvst.11.7.14

**Published:** 2022-07-18

**Authors:** Néstor Ventura-Abreu, Marc Biarnés, Sofia Batlle-Ferrando, María Teresa Carrión-Donderis, Rafael Castro-Domínguez, María Jesús Muniesa, Elena Millá, Javier Moreno-Montañés, Marta Pazos

**Affiliations:** 1 Ophthalmology Institute, Hospital Clínic Barcelona, Barcelona, Spain; 2 Hospital Quirón-Teknon, Institut de la Màcula, Barcelona, Spain; 3 Ophthalmology Department, Clínica Universidad de Navarra, Pamplona, Spain

**Keywords:** optical coherence tomography, retinal imaging, glaucoma diagnosis, risk calculators

## Abstract

**Purpose:**

To clinically validate the diagnostic ability of two optical coherence tomography (OCT)-based glaucoma diagnostic calculators (GDCs).

**Methods:**

We conducted a retrospective, consecutive sampling of 76 patients with primary open-angle glaucoma, 107 glaucoma suspects, and 67 controls. Demographics, reliable visual field testing, and macular and optic disc OCT were collected. The reference diagnosis was compared against the probability of having glaucoma obtained from two GDCs derived from multivariate logistic regressions using quantitative and qualitative (GDC1) or only quantitative (GDC2) OCT data. The discrimination (area under the curve [AUC]) and calibration (calibration plots) were compared for both calculators and the best OCT parameters.

**Results:**

GDC2 was able to identify 46.9% more suspects and 14.7% more glaucomatous eyes than GDC1. Both GDCs obtained the highest discriminative ability in glaucomatous eyes (GDC1 AUC = 0.949; GDC2 = 0.943 vs inferior peripapillary retinal nerve fiber layer [pRNFL] = 0.931; *P* = 0.43). The discriminating ability was not as good for glaucoma suspects, but the GDCs were not inferior to pRNFL (GDC 1 AUC = 0.739; GDC2 = 0.730; inferior pRNFL = 0.760; *P* = 0.54) and GDC2 was still able to correctly identify up to 30.8% more cases than the conventional OCT classification. Calibration showed risk underestimation for both groups and calculators, but it was better in GDC2 and in patients with glaucoma.

**Conclusions:**

OCT-based calculators showed an excellent diagnostic performance in glaucomatous eyes. GDC2 was able to identify approximately 30% more cases than the conventional pRNFL inferior OCT classification in both groups, suggesting a potential role of these composite scores in clinical practice.

**Translational Relevance:**

These OCT-based calculators may improve glaucoma diagnosis in clinical care.

## Introduction

Glaucoma-related visual impairment and blindness are preventable with timely diagnosis and treatment.^1^ The current standard diagnosis is based on clinical examination, considering both the characteristic appearance of the optic nerve head (ONH) and its associated functional deterioration obtained by visual fields. However, even among glaucoma specialists, the interobserver agreement to determine these changes is fair to moderate at best.^2^^,^^3^

In its early stages, glaucoma already causes retinal ganglion cells axons and subsequently their bodies, to die. The advances in optical coherence tomography (OCT) allow the accurate quantification of this thinning at the optic disc (ONH parameters and peripapillary retinal nerve fiber layer [pRNFL]) and macular regions (ganglion cell complex). Even though pRNFL parameters slightly outperform the ganglion cell–inner plexiform layer (GCIPL) for glaucoma diagnosis, the analysis of individual parameters from the OCT has shown a noteworthy discriminative accuracy.^4^^,^^5^ Lately, several combinations of these parameters have proven to improve the diagnostic ability of isolated OCT parameters, especially in early glaucoma.^6^^,^^7^

The glaucoma diagnostic calculators (GDCs) are two composite scores designed by the Network of Spanish Glaucoma Program from the Red Temática de Investigación Corporativa (RETIC) that use combinations of different Cirrus OCT (Carl Zeiss Meditec, Inc., Dublin, CA) parameters. These GDCs were tested in patients with stablished and preperimetric glaucoma, showing excellent and good discrimination from healthy controls.^8^^,^^9^ The purpose of the current research is two-fold: to externally validate the discriminative ability of these two GDCs and to compare it with that of isolated OCT parameters.

## Methods

### Study Design

This retrospective cohort study included consecutive patients attending the glaucoma and ophthalmology primary care departments at Hospital Clínic (Barcelona, Spain) from June 2019 to June 2021. The ethics committee of our institution approved the study. A waiver of written informed consent was granted owing to the retrospective design and because the data were collected from regular clinical practice. The study adhered to the tenets of the Declaration of Helsinki.

Inclusion criteria were patients aged 18 years or older, spherical equivalent of ±5.0 diopters (D), astigmatism of ±3.0 D, best-corrected visual acuity of 20/40 or better, and normal open-angle on gonioscopy. Exclusion criteria were corneal or retinal pathologies (including drusen), amblyopia, systemic diseases, or neurologic disorders that could affect test results, and intraocular surgery other than uncomplicated phacoemulsification 6 months before the examination. One or both eyes of each participant could be included according to the eligibility criteria described.

All participants underwent a complete ophthalmic examination, with visual acuity, pachymetry, slit-lamp biomicroscopy of the anterior and posterior segments, Goldmann applanation tonometry, gonioscopy, 24-2 SITA standard visual field testing (VFT) (Humphrey Field Analyzer; Carl Zeiss Meditec, Dublin, CA), and RNFL, optic disc, and GCIPL analysis using Cirrus HD-OCT. Based on clinical examination by two glaucoma specialists (M.P., N.V.A.), patients were classified into three groups: controls, glaucoma suspects, and those with glaucoma.^10^ Healthy controls had a normal ONH appearance, an intraocular pressure (IOP) of less than 21 mm Hg, and a normal VFT. Glaucomatous eyes had to have elevated untreated IOP of greater than 21 mm Hg, an abnormal optic disc appearance (thinning of the neuroretinal rim, notches, or papillary hemorrhages), or RNFL defects, with a correspondent and reproducible visual field defect as described by Anderson et al.^11^ Glaucoma suspects included individuals with an elevated IOP and/or glaucomatous optic disc appearance but without functional defects detectable with VFT.^12^

### Visual Field and OCT Acquisition and Analysis

VFT and OCT examinations were performed on the same day by experienced optometrists. All patients had reliable VFT with fixation losses, false positives (FP), and false negatives of less than 25%. The OCT signal strength was 6 or higher without misalignments, projection artifacts, or errors in the segmentation. OCT volume scans centered on the optic disc and the macula were acquired and analyzed and ONH, pRNFL, and GCIPL parameters were collected. Their color-code classification from the reference database was also recorded: red (<1st percentile), yellow (1st–5th percentile), and green (5th–95th percentile).

### Glaucoma Diagnosis Calculators

The RETICs GDCs details have been previously reported.^8^^,^^9^ Briefly, two predictive models from multivariate logistic regression were tested in 500 eyes and validated in 187 eyes of different patients from the same sites. The values used to develop each GDC were derived exclusively from the HD-OCT Cirrus, using a combination of both qualitative (color-coding classification) and quantitative data (ONH, pRNFL, and GCIPL measurements; GDC1), or only quantitative data (GDC2). The selected parameters were those who had the best discriminative ability: inferior pRNFL, inferior–temporal GCIPL, and average and vertical cup/disc ratios values (quantitative data); and superior–nasal, superior–temporal, and minimum GCIPL, and average cup/disc ratio color-coded classification (qualitative data). Both calculators provide a probability of having glaucoma with results ranging from 0% to 100% and classify the predicted probability of a glaucoma diagnosis in three categories: low (<30%), intermediate (30%–60%), and high (>60%).

### Statistical Analyses

The baseline characteristics of participants were described using the mean (standard deviation) or the median (interquartile range) for quantitative and number (percentage) for categorical variables. To determine if parametric (mean, standard deviation) or nonparametric (median, interquartile range) measurements were reported, graphical (standardized normal probability plots) and statistical (Shapiro–Wilk) methods were used to evaluate the distribution for each quantitative variable separately. Their characteristics were compared between groups defined by glaucoma status using either analysis of variance independent *t* test, Mann–Whitney, or Fisher exact tests, as appropriate.

The ability of the Cirrus OCT parameters to discriminate between healthy and glaucomatous eyes was tested separately for the binary and the continuous variables generated by the instrument. Regarding binary variables, the color scale provided by the sector map was classified as follows: the color “green” was considered normal, whereas “yellow” and “red” were considered abnormal. The following measures were calculated for each disc, pRNFL, and macular GCIPL color scales: sensitivity, specificity, positive and negative predictive value, and likelihood ratios positive and negative. For the quantitative variables, areas under the curve (AUC) adjusted for potential confounders (those differences between baseline features not related to the IOP or the visual field) were determined. The parameters with the highest AUC for each category (ONH, pRNFL, and GCIPL) for differentiating healthy from glaucoma eyes were formally compared and receiver operating characteristic curves were generated. For the GDCs discriminative performance evaluation, the clinical diagnosis was the reference test, and the results from each GDC were considered the test. The diagnostic ability of each GDC was tested in two situations: healthy controls versus glaucoma suspects and healthy controls versus glaucomatous eyes. The following performance measures were obtained for each GDC/best individual categorical parameter and subgroup: sensitivity, specificity, positive predictive value, negative predictive value, likelihood ratio positive, and likelihood ratio negative. To determine which GDC provided the best results in each subgroup, their AUCs were compared. Venn diagrams were provided to compare the identification of glaucoma suspects and glaucomatous eyes with the best OCT parameter and each GDC. Finally, calibration (the agreement between the GDC predicted probability of glaucoma and its actual, observed probability in groups defined by their probability of disease) was evaluated through calibration plots.

Stata IC version 15.1 (StataCorp LLC; College Station, TX) was used to analyze the data. A two-tailed *P* value of less than 0.05 was considered statistically significant.

## Results

We included 250 eyes from 194 patients. There were 98 females (50.5%), the mean age was 67.5 years ± 11.2, and all were of European descent. The demographic characteristics are shown in Table 1.

**Table 1.** Baseline Characteristics of Participants in the Study

	All	Control	Suspect	Glaucoma	*P* Value
No. of patients (eyes)	194 (250)	48 (67)	89 (107)	57 (76)	NA
Female sex	98 (50.5)	33 (68.8)	39 (43.8)	26 (45.6)	0.01
Age, years	67.5 (11.2)	61.0 (11.3)	67.1 (10.2)	73.6 (9.5)	<0.0001
IOP, mm Hg^*^	22.6 (4.8)	16.5 (3.0)	24.0 (3.5)	24,0 (3.0)	0.0001
MD, dB^*^	−1.93 (2.79)	−0.12 (1.50)	−0.70 (1.39)	−4.85 (3.15)	0.0001
VFI^*^	96.4 (5.8)	100 (1.0)	99 (2.0)	93.0 (9.0)	0.0001
CCT, µm	545.3 (37.4)	549.8 (36.7)	550.2 (37.2)	533.6 (36.4)	0.01
SStrength, papillary	7.6 (1.0)	7.9 (1.1)	7.6 (1.0)	7.3 (0.9)	0.003
SStrength, macula	8.4 (1.1)	8.7 (0.9)	8.5 (1.0)	7.9 (1.1)	<0.0001

CCT, central corneal thickness; MD, mean deviation in the visual field; NA, not applicable; SStrength, signal strength; VFI, visual field index.

* Medians (interquartile range) are reported owing to non-normal distribution; otherwise, the mean (standard deviation) is reported.

Values are mean (standard deviation) for quantitative and number (%) for categorical variables.

Regarding the isolated OCT parameters performance, we analyzed the ability of the color (binary) scales of each sector map to discriminate between healthy and glaucoma suspects, and between healthy controls and patients with glaucoma (Supplementary Tables S1 and S2). The results generally showed very low sensitivity (<25%) and high specificity (>80%) in the healthy versus glaucoma suspects comparison, and moderate sensitivity and high specificity (>80%), between healthy and patients with glaucoma, particularly for pRNFL and GCIPL parameters (specificity of >90%). The pRNFL Inferior thickness obtained the best results for glaucoma and suspects (AUC = 0.931 and 0.760, respectively) followed by average pRNFL (0.925 and 0.745) and minimum GCIPL (0.919 and 0.735), although differences were not statistically significant. For the ONH measurements, the best parameter was the vertical cup-to-disc ratio (AUC = 0.916 and 0.727). Details of the other OCT parameters are available in Supplementary Tables S3 and S4.

**Figure 1.**
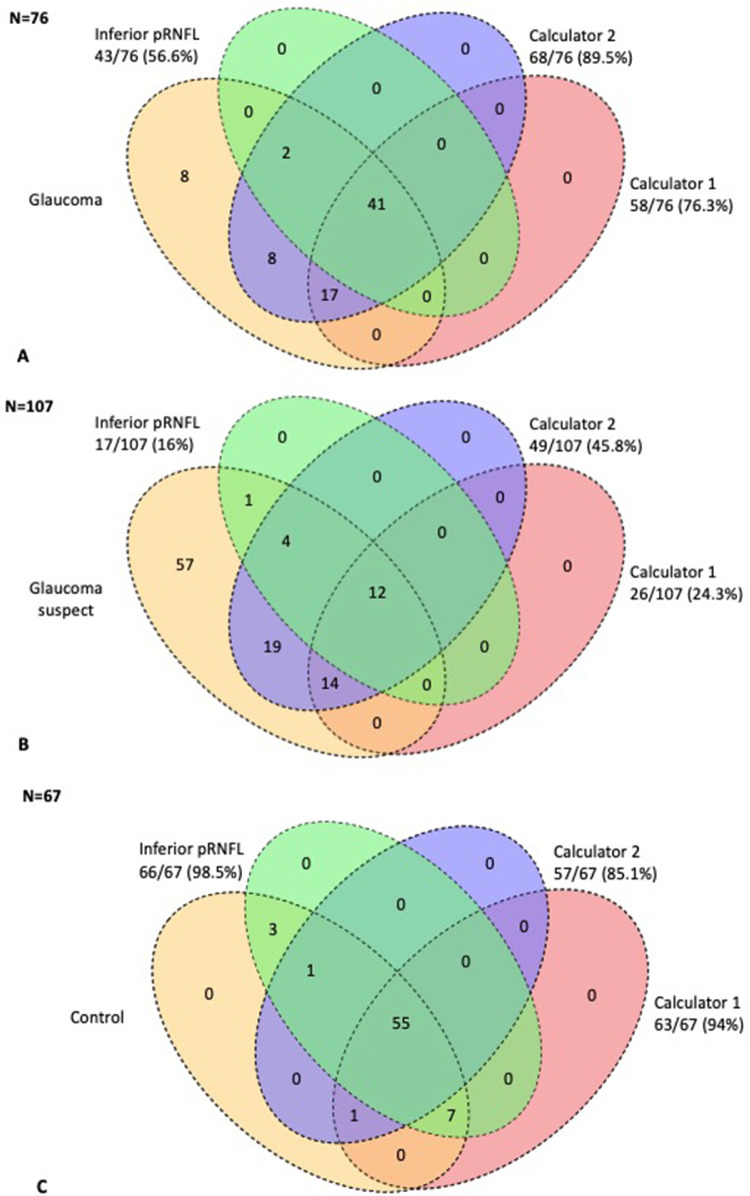
Venn diagrams comparing the number of eyes identified by the four diagnostic systems in each group: Clinical examination (*yellow*), inferior pRNFL (*green*), calculator 1 (*red*) and calculator 2 (*purple*). *Top,* Venn diagrams of the glaucomatous eyes (A). *Middle,* Venn diagrams of the glaucoma suspects (B). *Bottom,* Venn diagrams of the number of controls classified by each diagnostic system (C). The reference test or gold standard in each case was the clinical examination. N, number of eyes in each group; pRNFL, peripapillary retinal nerve fiber layer.

The discriminative ability measures for both calculators are displayed in Table 2 and additionally for each individual categorical (sector) parameter in Supplementary Table S5. The sensitivity for the detection of glaucoma suspects was rather low and ranged from 24.3% to 45.8% with GDC1 and GDC2, respectively. Accordingly, the positive predictive value was close to 90% in all cases. These values increased to 76.3% and 89.5% for the detection of established glaucoma. In contrast, the specificity was good to excellent, ranging from 85.1% to 94.0%. The likelihood ratio positive was particularly high for the detection of glaucoma with GDC1 (12.80) and to discard the disease with GDC2 (likelihood ratio negative = 0.12). When evaluating the AUCs, we found a moderate ability for the calculators to differentiate between controls and glaucoma suspects (GDC1 AUC = 0.739; GDC2 AUC = 0.730) and a high discriminative ability between controls and glaucoma patients (GDC1 AUC = 0.949; GDC2 AUC = 0.943). There were no statistically significant differences when comparing the diagnostic performance of GDC1 and GDC2 in the different groups (see Supplementary Table S6). However, using the greater than 30% cutoff point (intermediate and high probability) to compare the agreement between both calculators, all eyes correctly identified by GDC1 were also identified by GDC2, irrespective of the group. In addition, GDC2 was able to identify 46.9% more cases with suspect glaucoma and 14.7% more with established glaucoma than GDC1 (Figs. 1A and 1B).

**Table 2.** Discriminating Parameters of Each Calculator for Each Subgroup Being Compared

Calc	Subgroup	Sens, %	Spec, %	PPV, %	NPV, %	LR+	LR–	AUC
*1*	Suspects	24.3 (16.5–33.5)	94.0 (85.4–98.3)	86.7 (69.3–96.2)	43.8 (35.5–52.3)	4.07 (1.49–11.1)	0.81 (0.71–0.91)	0.739 (0.664–0.814)
	Glaucoma	76.3 (65.2–85.3)	94.0 (85.4–98.3)	93.5 (84.3–98.2)	77.8 (67.2–86.3)	12.80 (4.90–33.3)	0.25 (0.17–0.38)	0.949 (0.916–0.982)
*2*	Suspects	45.8 (36.1–55.7)	85.1 (74.3–92.6)	83.1 (71.0–91.6)	49.6 (40.1–59.0)	3.07 (1.67–5.63)	0.64 (0.52–0.78)	0.730 (0.654–0.805)
	Glaucoma	89.5 (80.3–95.3)	85.1 (74.3–92.6)	87.2 (77.7–93.7)	87.7 (77.2–94.5)	5.99 (3.37–10.7)	0.12 (0.06–0.24)	0.943 (0.906–0.980)

AUC, area under the curve; Calc, calculator; LR, likelihood ratio; NPV, negative predictive value; PPV, positive predictive value; Sens, sensitivity; Spec, specificity.

Values in parentheses represent 95% confidence intervals.

**Figure 2.**
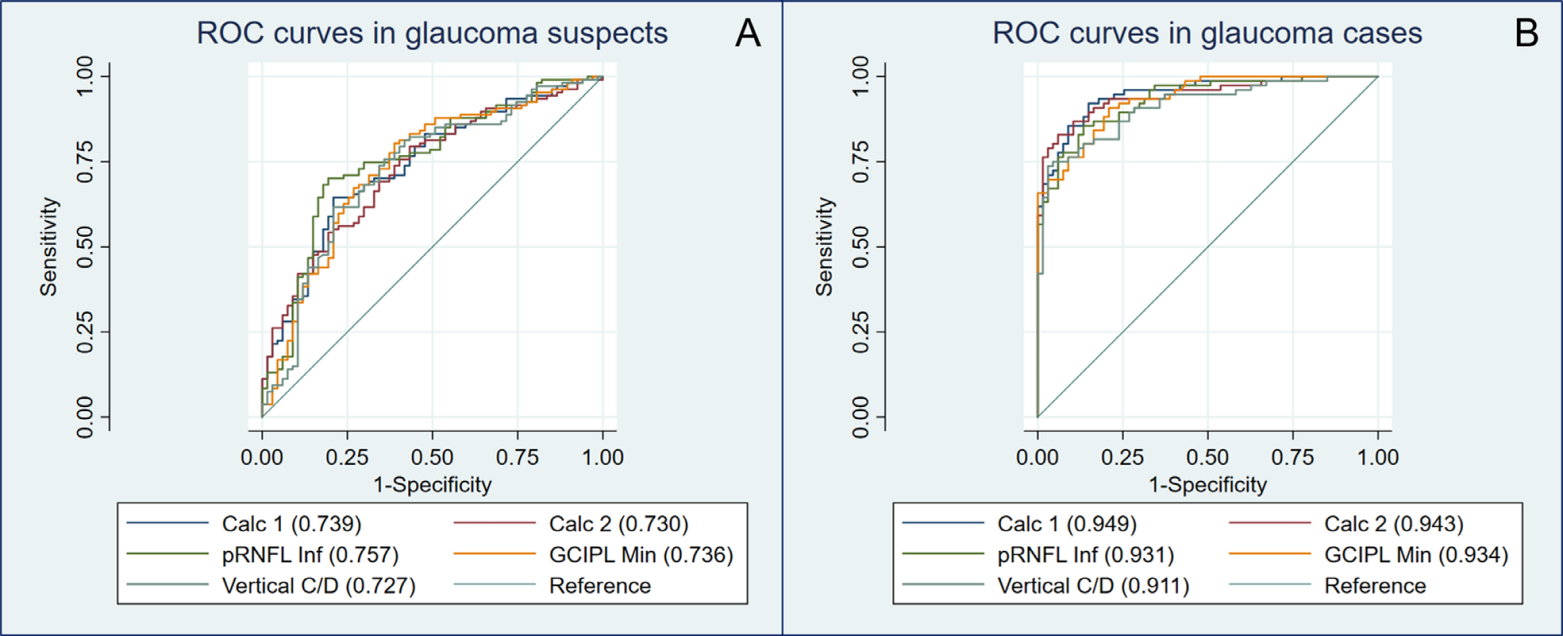
Receiver operating characteristic (ROC) curves and the corresponding areas under the ROC curve (AUC) of the best single OCT parameters and calculators 1 and 2, for the glaucoma suspects set of patients (A) and the glaucoma cases group (B). Calc 1, GDC 1; Calc 2, GDC 2; C/D, cup/disc ratio; pRNFL Inf, pRNFL, inferior sector.

We compared the diagnostic performance of the best OCT parameters with the one obtained with both GDCs in glaucoma suspects and glaucoma cases (Supplementary Table S7). For glaucoma suspects, the calculators were not able to overcome inferior pRNFL (GDC 1 AUC = 0.739; GDC2 AUC = 0.730; inferior pRNFL AUC = 0.760; *P* = 0.64) (Fig. 2A). However, when looking at the Venn diagram comparison, we found that GDC2 was able to correctly identify 30.8% more cases than the more conventional pRNFL inferior parameter OCT classification (Fig. 1B).

In established glaucoma, both GDCs obtained higher discriminative ability (GDC1 AUC = 0.949; GDC2 = 0.943; inferior pRNFL = 0.931), but differences were not statistically significant (*P* = 0.43) (Fig. 2B). Again, we found that GDC2 was able to correctly identify 89% of glaucoma cases, whereas inferior pRNFL only classified 56% of them (all of them also identified by GDC2) (Fig. 1A).

When applied to the 67 controls, inferior pRNFL correctly classified 66 cases (98.5%), whereas GDC1 and GDC2 missed 4 (6.0%) and 10 (14.9%) cases, respectively (Fig. 1C).

**Figure 3.**
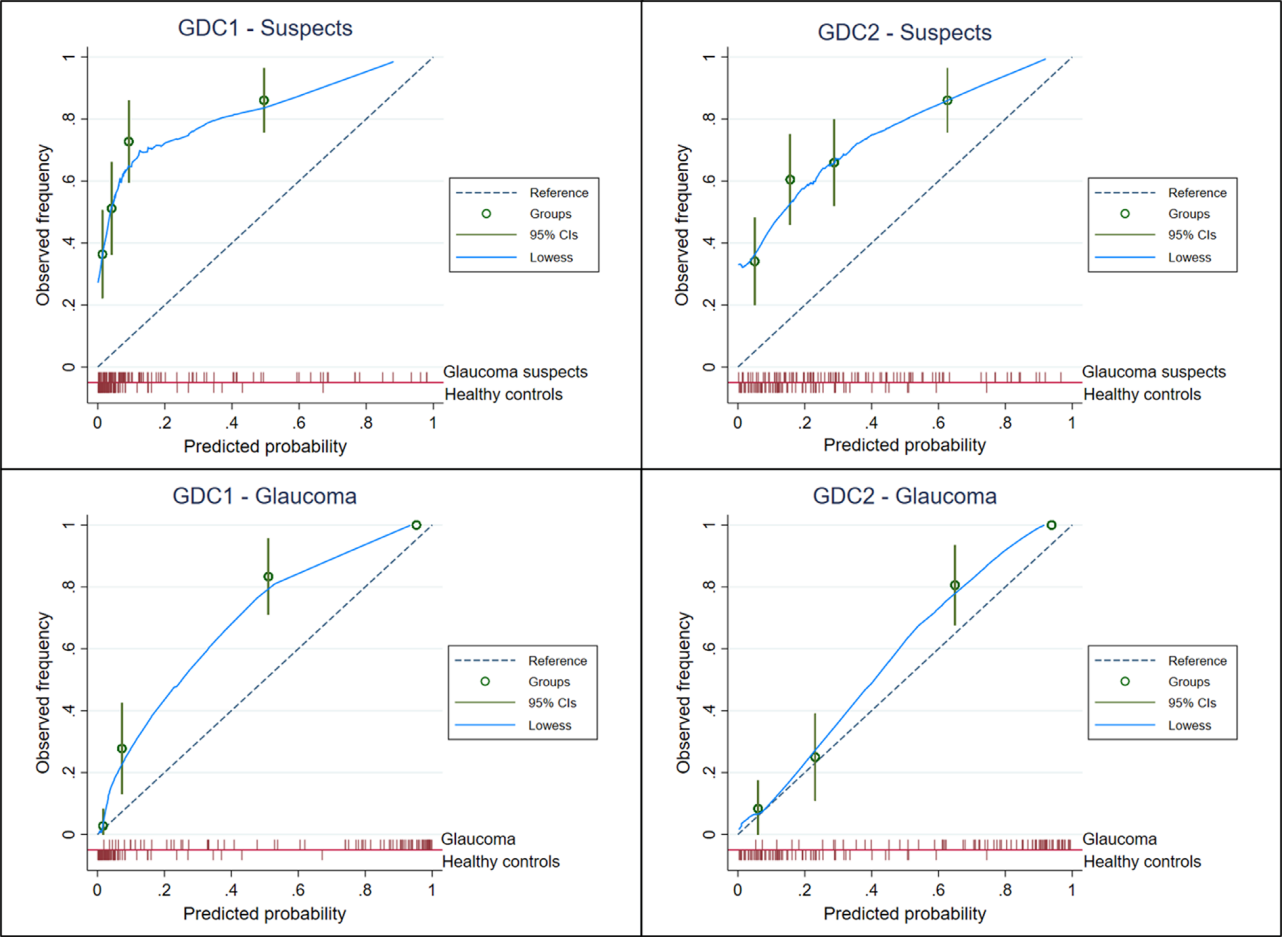
Calibration plots. *Top left*, calibration for GDC1 in glaucoma suspects. *Top right*, calibration for GDC2 in the same patients. *Bottom left*, calibration of GDC1 for patients with glaucoma. *Bottom right*, calibration of GDC2 for glaucoma. Overall, calibration was suboptimal because of marked risk underestimation, better for GDC2 as compared with GDC1 and for identification of glaucoma than for disease suspects.

Calibration plots are shown in Figure 3. The calibration of the GDC1 for glaucoma suspects shows a marked underestimation of risk (ratio of expected to observed cases of 0.26 or 26%), with a calibration in the large (CITL) of 3.32 and a slope of 0.56. Similar overall behavior but with modestly better numerical results was shown for GDC2 in the same scenario, with a ratio of expected to observed cases of 0.45, CITL of 1.96, and slope of 0.59.

Nonetheless, both calculators performed better in glaucoma cases. GDC1 had an ratio of expected to observed cases of 0.73, CITL of 1.62, and a slope of 1.20, whereas the values for GDC2 were 0.89, 0.52, and 1.49, respectively. Overall, these results imply that both GDCs underestimate the risk of these events (more so for glaucoma suspects than for glaucoma eyes), which is particularly marked for the use of GDC1 in glaucoma suspects. In contrast, calibration was good in GDC2 when used in glaucomatous eyes.

## Discussion

In the era of precision medicine, diagnostic calculators are a good option to personalize the risk of developing a certain disease. In ophthalmology, there are several examples related to different conditions, such as retinopathy of prematurity^13^ or diabetic retinopathy, that allow both risk stratification and custom-made frequency of the screening visits that may ultimately decrease health costs.^14^^,^^15^ However, after the development and validation of the risk estimation calculators using the results of the Ocular Hypertension Treatment Study and the European Glaucoma Prevention Study, glaucoma has been the front runner in its clinical application. ^16^^,^^17^ Although new biomarkers may modify the original interpretation,^18^ currently its use has still a role as a supplementary tool to help decide which patients would benefit the most from treatment.^19^

Glaucoma diagnosis remains challenging, especially in the early stages during which visual field defects are scarce or even absent.^20^ In these situations, structural changes in the pRNFL and GCIPL have shown an excellent diagnostic ability. More recently, the best outcomes have been reported when combining ONH, pRNFL, and GCIPL parameters,^6^ as it happens in composite scores like the ones obtained by GDCs.^8^^,^^9^ In our study, we aimed to clinically validate the diagnostic ability of two OCT-based GDCs comparing it with the one obtained by the conventional OCT parameters.

We first evaluated the diagnostic ability of isolated OCT parameters in our sample to see if they were comparable with the ones obtained in the RETICs development and validation studies. In their works, the best OCT parameters for the ONH, pRNFL, and macula were the vertical cup-to-disc ratio, inferior RNFL, and inferior–temporal GCIPL, respectively.^8^^,^^9^ Similar to their results, in our sample the best OCT discriminative ability was achieved by inferior pRNFL and the best ONH parameter was the vertical cup-to-disc ratio. Interestingly, although our AUCs were comparable, our best macular parameter was the minimum GCIPL. These differences may be due to the different range of glaucoma severities, but in line with our results, previous works have shown the minimum GCIPL to achieve better glaucoma diagnostic performance than the other macular parameters at comparable specificities.^21^ As expected, we also found that the OCT parameters had better diagnostic ability in glaucoma cases compared with glaucoma suspects and that the diagnostic performance improved with disease severity.^22^

We then compared the GDCs performance in our cohort, to evaluate the utility of adding qualitative information based on a color-coded classification into the model (GDC1) versus using only quantitative data (GDC2). In this regard, both GDCs behaved very similarly, without statistically significant differences in suspects (AUC = 0.739 and 0.73; *P* = 0.56) or glaucomatous eyes (AUC = 0.949 and 0.943; *P* = 0.61). However, when comparing the Venn diagrams, GDC2 was able to identify 46.9% and 14.7% more cases in the glaucoma suspects and glaucoma groups than GDC1, respectively, suggesting that adding qualitative information may not improve the model performance. Several reasons could explain this: first, color coding is built using a normative database that still can lead to a moderate FP rate.^23^ Second, the FP rate can decrease when the classification is adapted considering coexisting factors.^24^ And third, the selection of parameters to create a composite score can be challenging: choosing different indicators from the same source and with analogous behavior can lead to multicollinearity, where many variables contain similar information that is, therefore, redundant. In this situation principal component analyses^7^ or unit-weighted composite scores would be more suitable,^25^ and fewer properly selected variables would be probably more precise as well.^26^

After that, we compared the GDCs and standard OCT parameters AUCs. In manifest glaucoma, both GDCs obtained excellent diagnostic ability (GDC1 AUC = 0.949; GDC2 AUC = 0.943, inferior pRNFL = 0.931; *P* = 0.43). In contrast, when used in glaucoma suspects, GDCs AUCs were only modest and similar to pRNFL parameters, and lower than the ones obtained in the RETICs preperimetric glaucoma study.^9^ These differences may be due to several reasons: first, our glaucoma suspects included ocular hypertensives; and second, in their inclusion criteria they used OCT parameters, possibly overestimating the calculators' diagnostic performance.

When considering classification cases of glaucoma and suspects with the different diagnostic systems (Fig. 1), we found that at the same AUC levels, GDC2 missed fewer glaucomatous patients and suspects compared with inferior pRNFL (10.5% vs 43.5% false negatives for glaucoma and 84% and 54% false negatives for suspects, respectively), but at the expense of having significantly more FPs (15.0% vs 1.5%). In a glaucoma clinical context, early diagnosis is very important; therefore, false negatives are possibly more concerning than FP. In this regard, although calculator 2 wrongly classifies 14% more controls than single parameters, it is able to diagnose 33% more glaucoma cases and 30% suspects that would have been overlooked if only the pRNFL color coding had been taken into account, which we believe may be of use in clinical practice. In contrast, GCD1 was much better detecting controls than GDC2 being almost as specific as inferior pRNFL (94.0% vs 98.5%, respectively), but with less sensitivity, so possibly both calculators are complementary and may be applicable in different clinical situations.

Taken together, these findings suggest a potential clinical application of this kind of calculators, especially in early or inconclusive cases, which are the most difficult to diagnose. It needs to be elucidated whether the incorporation of other clinical metadata like IOP, corneal thickness, or VFT into these algorithms could improve even more their discriminative ability.

Finally, an important and often under-rated aspect of external validation is calibration, the comparison between the predicted and observed disease risk. Despite very good discrimination, both calculators showed suboptimal calibration in both groups. All models generated CITL or intercepts of greater than 0 (ranging from 0.52 to 3.32), which imply an underestimation of risks, whereas slopes were of less than 1 for suspects (0.56 and 0.59) and greater than 1 for manifest glaucoma (1.20 and 1.49), meaning extreme (very wide) or constrained (very narrow) risk predictions, respectively.^27^ Therefore, model updating might be required, as extensively discussed by Steyerberg and Vergouwe.^28^

The present study has limitations. First, there are limitations inherent to the retrospective design. Second, the heterogeneous nature of the glaucoma suspects group in terms of structural characteristics may have underestimated the GDCs AUCs, as well as the relatively limited sample size. Third, including both eyes of some patients may have influenced our results, since the outcomes from two eyes within a participant tend to resemble more each other than outcomes from the eyes of other participants. Although this is particularly relevant during the GDCs predictive algorithm development and the main reason to include only one eye for each patient, it is not so important for the external clinical validation, because GDCs were designed to evaluate the risk of having glaucoma at the eye level and not at a patient level, and this is what is done in clinical practice. For this reason, and to keep real-world clinical conditions, no correction for between-eye correlation was applied. Last, we clinically validated the GDCs in a different population, but because it is also a Spanish, European-descent, and single-center cohort, the outcomes might not be fully generalizable.

In summary, in glaucomatous eyes, the RETICs OCT-based calculators showed the best diagnostic performance compared with glaucoma suspects. GDC2 was able to identify 33% more cases than the conventional inferior pRNFL classification in glaucomatous eyes; in glaucoma suspects, the discriminative ability was similar to inferior pRNFL, but GDC2 still detected 30.8% more cases that would have been missed with the conventional OCT classification. However, both calculators underestimated disease risk, particularly in glaucoma suspects and with the use of GDC1.

## Supplementary Material

Supplement 1
