# Supplementary material for: External Validation and Clinical Applicability of Two Optical Coherence Tomography–Based Risk Calculators for Detecting Glaucoma
Source: Transl Vis Sci Technol. 2022 Jul 18;11(7):14. doi: 10.1167/tvst.11.7.14 (PMC9308015; doi:10.1167/tvst.11.7.14)
Supplement: Supplement 1 [file tvst-11-7-14_s001.pdf]

Supplementary material

	Sens	Spec	PPV	NPV	LR+	LR-
Optic disc						
Rim area	**24.3 (16.5-33.5)**	86.6 (76-93.7)	74.3 (56.7-87.5)	41.7 (33.4-50.4)	1.81 (0.90-3.62)	0.87 (0.76-1.01)
Mean C/D	18.7 (11.8-27.4)	85.1 (74.3-92.6)	66.7 (47.2-82.7)	39.6 (31.5-48.1)	1.25 (0.63-2.51)	0.96 (0.84-1.09)
Vert C/D	23.4 (15.7-32.5)	89.6 (79.7-95.7)	78.1 (60-90.7)	42.3 (34-50.8)	2.24 (1.02-4.88)	0.86 (0.75-0.98)
Cup vol	18.7 (11.8-27.4)	82.1 (70.8-90.4)	62.5 (43.7-78.9)	38.7 (30.7-47.3)	1.04 (0.55-1.99)	0.99 (0.86-1.14)
Peripapilar retinal nerve fiber layer						
Average	21.5 (14.1-30.5)	**98.5 (92-100)**	**95.8 (78.9-99.9)**	**44.0 (35.9-52.3)**	**14.40 (1.99-104)**	**0.80 (0.72-0.88)**
Sup	19.6 (12.6-28.4)	95.5 (87.5-99.1)	87.5 (67.6-97.3)	42.7 (34.6-51)	4.38 (1.36-14.1)	0.84 (0.76-0.94)
Inf	15.9 (9.5-24.2)	**98.5 (92-100)**	94.4 (72.7-99.9)	42.3 (34.4-50.5)	10.6 (1.45-78.1)	0.85 (0.78-0.93)
Nas	0.9 (0.0-5.1)	**98.5 (92-100)**	50.0 (1.26-98.7)	38.4 (31.1-46.1)	0.63 (0.04-9.84)	1.01 (0.97-1.04)
Temp	9.4 (4.6-16.5)	94.0 (85.4-98.3)	71.4 (41.9-91.6)	39.4 (31.8-47.4)	1.57 (0.51-4.79)	0.96 (0.89-1.05)
Ganglion cell-inner plexiform layer						
Average	12.1 (6.6-19.9)	97.0 (89.6-99.6)	86.7 (59.5-98.3)	40.9 (33.2-48.9)	4.07 (0.95-17.5)	0.91 (0.83-0.98)

Minimum	16.8 (10.3-25.3)	97.0 (89.6-99.6)	90.0 (68.3-98.8)	42.2 (34.3-50.4)	5.64 (1.35-23.5)	0.86 (0.78-0.94)
Sup Temp	15.9 (9.5-24.2)	94.0 (85.4-98.3)	81.0 (58.1-94.6)	41.2 (33.3-49.4)	2.66 (0.94-7.57)	0.90 (0.81-0.99)
Sup	15.9 (9.5-24.2)	**98.5 (92.0-100)**	94.4 (72.7-99.9)	42.3 (34.4-50.5)	10.6 (1.45-78.1)	0.85 (0.78-0.93)
Sup Nas	10.3 (5.2-17.7)	95.5 (87.5-99.1)	78.6 (49.2-95.3)	40.0 (32.3-48.0)	2.30 (0.67-7.93)	0.94 (0.87-1.02)
Inf Temp	15.0 (8.8-23.1)	**98.5 (92.0-100)**	94.1 (71.3-99.9)	42.0 (34.2-50.2)	10.00 (1.36-73.8)	0.86 (0.79-0.94)
Inf	14.0 (8.1-22.1)	**98.5 (92.0-100)**	93.8 (69.8-99.8)	41.8 (34.0-49.9)	9.39 (1.27-69.5)	0.87 (0.80-0.95)
Inf Nas	10.3 (5.2-17.7)	**98.5 (92.0-100)**	91.7 (61.5-99.8)	40.7 (33.1-48.7)	6.89 (0.91-52.1)	0.91 (0.85-0.98)

Supplementary table 1. Discrimination indices between healthy controls and glaucoma suspects for optic disc, peripapillary retinal nerve fiber layer and ganglion cell-inner plexiform layer measurements. Values for Sens, Spec, PPV and NPV are percentages and for LR+/-, ratios. Best values for each parameter shown in bold. C/D: cup-to-disc ratio; Inf: inferior; LR+/-: likelihood ratio positive and negative, respectively; Nas: nasal; NPV: negative predictive value; PPV: positive predictive value; Sens: sensitivity; Spec: specificity; Sup: superior; Temp: temporal; Vert: vertical; Vol: volume.

	**Sens**	**Spec**	**PPV**	**NPV**	**LR+**	**LR-**
Optic disc						
Rim area	**63.2 (51.3-73.9)**	86.6 (76.0-93.7)	84.2 (72.1-92.5)	67.4 (56.5-77.2)	4.70 (2.50-8.84)	0.43 (0.31-0.58)
Mean C/D	57.9 (46.0-69.1)	85.1 (74.3-92.6)	81.5 (68.6-90.7)	64.0 (53.2-73.9)	3.88 (2.12-7.09)	0.50 (0.37-0.66)
Vert C/D	61.8 (50.0-72.8)	89.6 (79.7-95.7)	87.0 (75.1-94.6)	67.4 (56.7-77.0)	5.92 (2.87-12.2)	0.43 (0.32-0.57)
Cup vol	52.6 (40.8-64.2)	82.1 (70.8-90.4)	76.9 (63.2-87.5)	60.4 (49.6-70.5)	2.94 (1.69-5.12)	0.58 (0.44-0.75)
Peripapilar retinal nerve fiber layer						
Average	53.0 47.3-70.4)	**98.5 (92.0-100.0)**	97.8 (88.5-99.9)	68.0 (57.8-77.1)	39.70 (5.62-280)	0.41 (0.32-0.54)
Sup	`57.9 (46.0-69.1)	95.5 (87.5-99.1)	93.6 (82.5-98.7)	66.7 (56.3-76.0)	12.90 (4.21-39.70)	0.44 (0.34-0.58)
Inf	56.6 (44.7-67.9)	**98.5 (92.0-100)**	97.7 (88.0-99.9)	66.7 (56.5-75.8)	37.90 (5.37-268)	0.44 (0.34-0.57)
Nas	7.9 (3.0-16.4)	**98.5 (92.0-100)**	85.7 (42.1-99.6)	48.5 (39.9-57.2)	5.29 (0.65-42.8)	0.94 (0.87-1.0)
Temp	26.3 (16.9-37.7)	94.0 (85.4-98.3)	83.3 (62.6-62.2)	52.9 (43.6-62.2)	4.41 (1.59-12.2)	0.78 (0.68-0.91)
Ganglion cell-inner plexiform layer						
Average	51.3 (45.0-61.5)	97.0 (89.6-99.6)	95.1 (83.5-99.4)	63.7 (53.6-73.0)	17.20 (4.31-68.50)	0.50 (0.40-0.64)
Minimum	**63.2 (51.3-73.9)**	97.0 (89.6-99.6)	96.0 (86.3 -99.5)	**69.6 (59.5-79.0)**	21.20 (5.35-83.7)	**0.38 (0.28-0.51)**

Sup Temp	55.3 (43.4-66.7)	94.0 (85.4-98.3)	91.3 (79.2-97.6)	64.9 (54.6-74.4)	9.26 (3.50-24.50)	0.48 (0.37-0.62)
Sup	47.4 (35.8-59.2)	**98.5 (92.0-100)**	97.3 (85.8-99.9)	62.3 (52.3-71.5)	31.70 (4.47-225.0)	0.53 (0.43-0.66)
Sup Nas	34.2 (23.7-46.0)	95.5 (87.5-99.1)	89.7 (72.6-97.8)	56.1 (46.5-65.4)	7.64 (2.42-24.10)	0.69 (0.58-0.82)
Inf Temp	60.5 (48.6-71.6)	**98.5 (92.0-100)**	**97.9 (88.7-99.9)**	68.8 (58.5-77.8)	40.6 (5.75-286)	0.40 (0.30-0.53)
Inf	50.0 (38.3-61.7)	**98.5 (92.0-100)**	97.4 (86.5-99.9)	63.5 (53.4-72.7)	33.50 (4.73-237.0)	0.51 (0.41-0.64)
Inf Nas	36.8 (26.1-48.7)	**98.5 (92.0-100)**	96.6 (82.2-99.9)	57.9 (48.3-67.1)	**54.7 (3.45-177.0)**	0.64 (0.54-0.76)

Supplementary table 2. Discrimination indices between healthy controls and glaucoma eyes for optic disc, peripapillary retinal nerve fiber layer and ganglion cell-inner plexiform layer measurements. Values for Sens, Spec, PPV and NPV are percentages and for LR+/-, ratios. Best values for each parameter shown in bold. C/D: cup-to-disc ratio; Inf: inferior; LR+/-: likelihood ratio positive and negative, respectively; Nas: nasal; NPV: negative predictive value; PPV: positive predictive value; Sens: sensitivity; Spec: specificity; Sup: superior; Temp: temporal; Vert: vertical; Vol: volume.

	AUC	95% CI	p-value
Optic disc			
Vert C/D	0.727	0.647 – 0.806	0.85
Mean C/D	0.720	0.639 – 0.801	
Cup vol	0.719	0.640 – 0.799	
Rim area	0.717	0.636 – 0.798	
Disc area	0.713	0.631 – 0.793	
Peripapillary retinal nerve fiber layer			
Inf	0.760	0.685 – 0.834	0.27
Average	0.745	0.667 – 0.822	
Sup	0.739	0.662 – 0.816	
Nas	0.715	0.634 – 0.797	
Temp	0.711	0.629 – 0.792	
Ganglion cell-inner plexiform layer			
Minimum	0.735	0.656 – 0.813	0.64
Sup Temp	0.723	0.643 – 0.803	
Inf Temp	0.720	0.639 – 0.800	
Average	0.715	0.634 – 0.796	
Inf Nas	0.715	0.635 – 0.795	
Inf	0.715	0.633 – 0.796	
Sup	0.714	0.633 – 0.794	
Sup Nas	0.713	0.632 – 0.793	

Supplementary table 3. Comparison of the AUC values for discriminating between healthy controls and glaucoma suspects in different anatomic locations. The AUC are ranked from highest to lowest and statistically compared for each structure independently. Values adjusted for age, sex, and signal strength. AUC: area under the curve; CI: confidence interval.

	AUC	95% CI	p-value
Optic disc			
Vert C/D	0.916	0.864-0.967	0.04
Rim area	0.913	0.861-0.965	
Mean C/D	0.886	0.825-0.947	
Cup vol	0.884	0.821-0.947	
Disc area	0.832	0.755-0.910	
Peripapillary retinal nerve fiber layer			
Inf	0.931	0.887-0.975	0.04
Average	0.925	0.878-0.972	
Sup	0.918	0.870-0.966	
Nas	0.847	0.773-0.921	
Temp	0.834	0.760-0.907	
Ganglion cell-inner plexiform layer			
Minimum	0.919	0.873-0.964	0.09
Inf Temp	0.899	0.847-0.952	
Sup Temp	0.897	0.842-0.951	
Inf	0.883	0.825-0.941	
Average	0.875	0.815-0.936	
Sup	0.872	0.810-0.934	
Inf Nas	0.844	0.775-0.914	
Sup Nas	0.833	0.758-0.908	

Supplementary table 4. Comparison of the AUC values for discriminating between healthy controls and glaucoma eyes in different anatomic locations. The AUC are ranked from highest to lowest and statistically compared for each structure independently. Values adjusted for age, sex, pachymetry and signal strength. AUC: area under the curve; CI: confidence interval.

Parameter	Subgroup	Sens, %	Spec, %	PPV, %	NPV, %	LR+	LR-	AUC
Superior-nasal GCIPL (sector)	*Suspects*	11.2 (5.9-18.8)	92.5 (83.4-97.5)	70.6 (44.0-89.7)	39.5 (31.8-47.6)	1.50 (0.55-4.08)	0.96 (0.87-1.06)	0.519 (0.475-0.562)
	Glaucoma	6.6 (2.2-14.7)	97.0 (89.6-99.6)	71.4 (29-96.3)	47.8 (39.2-56.5)	2.2 (0.4-11)	0.96 (0.90-1.04)	0.519 (0.483-0.553)
Superior-temporal GCIPL (sector)	*Suspects*	16.8 (10.3-25.3)	92.5 (83.4-97.5)	78.3 (56.3-92.5)	41.1 (33.1-49.3)	2.25 (0.88-5.79)	0.90 (0.81-1.00)	0.547 (0.499-0.594)
	Glaucoma	56.6 (44.7-67.9)	92.5 (83.4-97.5)	89.6 (77.3-96.5)	65.3 (54.8-74.7)	7.58 (3.19-18)	0.47 (0.36-0.61)	0.746 (0.681-0.810)
Minimum GCIPL (sector)	*Suspects*	19.6 (12.6-28.4)	92.5 (83.4-97.5)	80.8 (60.6-93.4)	41.9 (33.8-50.3)	2.63 (1.04-6.64)	0.87 (0.77-0.98)	0.561 (0.511-0.61)
	Glaucoma	64.5 (52.7-75.1)	92.5 (83.4-97.5)	90.7 (79.7-96.9)	69.7 (59.0-79.0)	8.64 (3.66-20.4)	0.38 (0.28-0.52)	0.785 (0.722-0.848)
Average C/D (sector)	*Suspects*	29.0 (20.6-38.5)	77.6 (65.8-86.9)	67.4 (52.0-80.5)	40.6 (32.0-49.7)	1.29 (0.76-2.21)	0.92 (0.77-1.09)	0.533 (0.467-0.599)
	Glaucoma	65.8 (54.0-76.3)	77.6 (65.8-86.9)	76.9 (64.8-86.5)	66.7 (55.1-76.9)	2.94 (1.83-4.72)	0.44 (0.32-0.62)	0.717 (0.643-0.791)

Supplementary Table 5. Diagnostic values for the best individual peripapillary and macular spectral domain optical coherence tomography parameters for discrimination of glaucoma and glaucoma suspects from healthy controls. Values between brackets represent 95% confidence intervals. AUC: area under the curve; C/D: cup-to-disc; GCIPL: ganglion cell-inner plexiform layer; LR+/-: likelihood ratios positive and negative; NPV: negative predictive value; PPV: positive predictive value; Sens: sensitivity; Spec: specificity.

Calc	Subgroup	Sens, %	Spec, %	PPV, %	NPV, %	LR+	LR-
1	*Suspects*	24.3 (16.5-33.5)	94.0 (85.4-98.3)	86.7 (69.3-96.2)	43.8 (35.5-52.3)	4.07 (1.49-11.1)	0.81 (0.71-0.91)
	Glaucoma	76.3 (65.2-85.3)	94.0 (85.4-98.3)	93.5 (84.3-98.2)	77.8 (67.2-86.3)	12.80 (4.90-33.3)	0.25 (0.17-0.38)
2	*Suspects*	45.8 (36.1-55.7)	85.1 (74.3-92.6)	83.1 (71.0-91.6)	49.6 (40.1-59.0)	3.07 (1.67-5.63)	0.64 (0.52-0.78)
	Glaucoma	89.5 (80.3-95.3)	85.1 (74.3-92.6)	87.2 (77.7-93.7)	87.7 (77.2-94.5)	5.99 (3.37-10.7)	0.12 (0.06-0.24)

Supplementary table 6. Discriminating parameters of each Calculator for each subgroup being compared. Values in parentheses represent 95% confidence intervals. Calc: calculator; LR: likelihood ratio; NPV: negative predictive value; PPV: positive predictive value; Sens: sensitivity; Spec: specificity.

Subgroup	Parameter	AUC	95% CI	p-value
Suspects	*pRNFL Inf*	0.760	0.685 – 0.834	0.54
	GCIPL Min	0.735	0.656 – 0.813	
	Vert C/D	0.727	0.647 – 0.806	
	GDC1	0.739	0.664 – 0.814	
	GDC2	0.730	0.654 – 0.805	
Glaucoma	*pRNFL Inf*	0.931	0.887-0.975	0.43
	GCIPL Min	0.919	0.873-0.964	
	Vert C/D	0.916	0.864-0.967	
	GDC1	0.949	0.916 – 0.982	
	GDC2	0.943	0.906 – 0.980	

Supplementary table 7. Comparison of the AUC values for discriminating between healthy controls and glaucoma suspects, and between controls and glaucoma eyes, of the best single OCT parameters, and calculators 1 and 2. The age, sex, and signal strength-adjusted AUC for each parameter, its 95% CI and their statistical comparison within each structure (disc, pRNFL and GCIPL) and calculators are reported. AUC: area under the curve; CI: confidence interval. GDC: Glaucoma Diagnostic Calculator. GCIPL: ganglion cell-inner plexiform layer. pRNFL Inf: peripapillary Retinal Nerve Fiber Layer, Inferior sector. Vert C/D: vertical cup/disc ratio.
